# Supplementary material for: Optimal duration of the apnea test for determining brain death: Benefit of the short-term apnea test
Source: PLoS One. 2022 Jul 28;17(7):e0272086. doi: 10.1371/journal.pone.0272086 (PMC9333206; doi:10.1371/journal.pone.0272086)
Supplement: Attachment — Submitted filename: Response to Editors and Reviewers.docx [file pone.0272086.s001.docx]

June 23, 2022

Steven Eric Wolf, MD

Academic Editor

*The Public Library of Science (PLoS) ONE*

Dear Editor:

We wish to re-submit the attached manuscript titled “**Optimal Duration of the Apnea Test for Determining Brain Death: Benefit of the Short-Term Apnea Test**.” The manuscript ID is PONE-D-22-01880.

The manuscript has been rechecked and appropriate changes have been made in accordance with yours and the reviewers’ suggestions.

The responses to the comments have been prepared and attached herewith.

We thank you and the reviewers for your thoughtful suggestions and insights, which have enriched the manuscript and produced a better and more balanced account of the research. We hope that the revised manuscript is now suitable for publication in your journal.

Data cannot be shared publicly for confidentiality reasons. Data are available from the Institutional Review Board (IRB) of Ewha Womans University Mokdong Hospital (contact the committee by email: [irb-mok@eumc.ac.kr](mailto:irb-mok@eumc.ac.kr)) for researchers who meet the criteria for accessing confidential data.

Thank you for your consideration. We look forward to hearing from you.

Sincerely,

Kyung Sook Hong

E-mail: [hongks@ewha.ac.kr](mailto:hongks@ewha.ac.kr)

**1. Please ensure that your manuscript meets PLOS ONE's style requirements, including those for file naming.**

Response: We would like to thank the Editor for evaluating our manuscript and for the constructive comment. Please note that we have verified that our manuscript meets *PLOS ONE*'s style requirements, including those for file naming.

**2. Please amend your current ethics statement to address.**

Response: As per the Editor’s suggestion, we have added the following sentence to the revised manuscript:

“The requirement of informed consent was waived because of the retrospective design of the study.”

**3. We note that you have stated that you will provide repository information for your data at acceptance. Should your manuscript be accepted for publication, we will hold it until you provide the relevant accession numbers or DOIs necessary to access your data. If you wish to make changes to your Data Availability statement, please describe these changes in your cover letter and we will update your Data Availability statement to reflect the information you provide**

Response: Unfortunately, despite our data being collected anonymously, we cannot provide the raw data directly because of our institution's policy. However, if necessary, the data may be available in the following process. We have added the following statement to the cover letter:

“Data cannot be shared publicly for confidentiality reasons. Data are available from the Institutional Review Board (IRB) of Ewha Womans University Mokdong Hospital (contact the committee by email: [irb-mok@eumc.ac.kr](mailto:irb-mok@eumc.ac.kr)) for researchers who meet the criteria for accessing

confidential data.”

4. **In your Data Availability statement, you have not specified where the minimal data set underlying the results described in your manuscript can be found. PLOS defines a study's minimal data set as the underlying data used to reach the conclusions drawn in the manuscript and any additional data required to replicate the reported study findings in their entirety. All PLOS journals require that the minimal data set be made fully available.**

Response: Please note that we have added the following statement to the cover letter:

“Data cannot be shared publicly for confidentiality reasons. Data are available from the Institutional Review Board (IRB) of Ewha Womans University Mokdong Hospital (contact the committee by email: [irb-mok@eumc.ac.kr](mailto:irb-mok@eumc.ac.kr)) for researchers who meet the criteria for accessing

confidential data.”

***Responses to reviewers’ comments***

The authors would like to thank the reviewers for their constructive critique to improve the manuscript. We have made every effort to address the issues raised and to respond to all comments. The revisions are indicated in red font in the revised manuscript. Please, find next a detailed, point-by-point response to the reviewers’ comments. We hope that our revisions would meet the reviewers’ expectations.

**Reviewer #1**

**1. Clarify in the result section and Table #1, when describing most common causes of brain death "Traumatic hemorrhage" and "Non traumatic hemorrhage". Please clarify that these are intracranial hemorrhages or describe**

Response: We would like to thank the reviewer for evaluating our manuscript and for the insightful comment. Please note that both “traumatic hemorrhage” and “non-traumatic hemorrhage” are intracranial hemorrhages. As per the reviewer’s suggestion, we have added the relevant information to Table 1.

**2. Unclear if within 5 minutes all patients had a PaCO2 above 60 mmHg. If that is the case, state that all patients achieve this goal. If not, stare the percent that did.**

Response: We would like to thank the reviewer for the kind comments to improve our manuscript. To convey accurate information to our readers, we have added relevant contents, as per the reviewer’s comments. Additionally, the positive criterion for apnea test in Korea is a PaCO_2_ level of ≥50 mmHg. Therefore, PaCO_2_ levels >60 mmHg were not observed in all patients. As per the reviewer’s suggestion, we have added the following sentence to the revised manuscript:

“In this study, the PaCO_2_ level exceeded 60 mmHg within 5 min in 76 (88.4%) and 81 (94.2%) cases of the 1^st^ and 2^nd^ apnea tests, respectively.”

**Reviewer #2**

**1. A standard time interval for the apnea test has been suggested by national societies but not formalized by the international medical community. This paper is an attempt at starting that process, but because of varying ethical and legal standards, one paper will not complete the process. The scientific and statistical methods used by the authors are sound and are supported by the provided data. The choice of the smallest time interval that is satisfactory from a PaCO2 endpoint for protection of organs for transplantation is understandable but may be hard to achieve consensus. The additional time suggested by the American Academy of Neurology should not incur too great an oxygen debt or acidemia. As shown by this paper, the hemodynamics are usually easily controlled with vasoactive infusions. Some patients will in fact have intact brainstem reflexes that were not appreciated until the apnea test. Before the patient is declared dead, physicians still owe them a fiduciary duty. As suggested in this paper, future work should also focus on standardizing the balance of the protocol of the apnea test. The presented protocol is reasonable if the additional time is added.**

Response: We would like to thank the reviewer for evaluating our manuscript and for his/her insightful comment. In the conclusion, we have stated that the appropriate duration of the apnea test is approximately 5 min. There are various debates concerning the duration of the apnea test for determination of brain death in Korea. As per the reviewer’s comments, we agree that the determination of brain death must be accompanied by ethical considerations. Therefore, as a further study, we will investigate the status of apnea tests in various institutions of Korea and plan ethical and reasonable suggestions for the positive criteria for apnea tests, including duration.
